# 

**Published:** 1882-03

**Authors:** 


### CONTENTS.

### *MISCELLANEOUS.*

| | PAGE. |
|---|---|
| Latent Medication. P. P. Wells, M. D., Brooklyn, N. Y., | 105 |
| Misrepresentation. Ad. Lippe, M. D., Philadelphia, | 110 |
| A Law of Nature. Wm. Jefferson Guernsey, M. D., Philadelphia, | 113 |
| Repetition of Dose. E. B. Nash, M. D., Cortland, N. Y., | 114 |
| The Homœopathic Medical Society of Central New York. C. P. Jennings, M. D., Secretary, | 116 |
| Pasteur's Experiments. R. R. Gregg, M. D., Buffalo, N. Y., | 121 |
| Vaccination. E. J. L., | 131 |
| A Gentle Hint, | 135 |
| American Joker. (Extract from.) | 136 |
| Table of Diarrhœa Symptoms, with Remedies. C. Hering, M. D., (Extract.) | 136 |

### *CLINICAL BUREAU:*

| | |
|---|---|
| Value of Clinical Reports. E. J. L., | 137 |
| Euthanasia in Phthisis. E. W. Berridge, M. D., London, | 138 |
| Clinical Reports, with Cases. Edward Rushmore, M. D., Plainfield, N. J., | 144 |
| PERISCOPE, | 146 |
| International Hahnemannian Association, | 152 |

